# Inhibitory Effect of *Nepeta deflersiana* on Climax Bacterial Community Isolated from the Oral Plaque of Patients with Periodontal Disease

**DOI:** 10.3390/molecules26010202

**Published:** 2021-01-03

**Authors:** Irfan Ahmad, Safia Irfan, Mohammed Abohashrh, Shadma Wahab, Shahabe Saquib Abullais, Mukhatar Ahmed Javali, Nazima Nisar, Mohammad Mahtab Alam, Saurabh Srivastava, Mohd Saleem, Gaffar Sarwar Zaman, Irshad Ahmad, Nasrin Mansuri

**Affiliations:** 1 Department of Clinical Laboratory Sciences, College of Applied Medical Sciences, King Khalid University, Abha 61421, Saudi Arabia; naznisar@gmail.com (N.N.); gaffarz@kku.edu.sa (G.S.Z.); nasrinmansuri17@gmail.com (N.M.); 2 Department of Physiology, College of Medicine, King Khalid University, Abha 61421, Saudi Arabia; safiaobaidur@gmail.com; 3 Department of Basic Medical Sciences, College of Applied Medical Sciences, King Khalid University, Abha 61421, Saudi Arabia; mabuhashra@kku.edu.sa (M.A.); mmalam@kku.edu.sa (M.M.A.); 4 Department of Pharmacognosy, College of Pharmacy, King Khalid University, Abha 61421, Saudi Arabia; shad.nnp@gmail.com; 5 Department of Periodontics and Community Dental Sciences, College of Dentistry, King Khalid University, Abha 61421, Saudi Arabia; drsaquib24@gmail.com (S.S.A.); jmahmad@kku.edu.sa (M.A.J.); 6 Department of Pharmaceutics, Era College of Pharmacy, Era University, Lucknow 226003, India; saurabhsrivastava.kgmu@gmail.com; 7 Department of Pathology, College of Medicine, University of Hail, Hail 2440, Saudi Arabia; m.saleem@uoh.edu.sa; 8 Department of Medical Rehabilitation Sciences, College of Applied Medical Sciences, King Khalid University, Abha 61421, Saudi Arabia; iabdulhamed@kku.edu.sa

**Keywords:** *N. deflersiana*, periodontal disease, red-complex bacteria, biofilm, antibacterial activity

## Abstract

Background: The red-complex bacteria are one of the most significant complexes found simultaneously in subgingival plaque next to the periodontal pocket. The current antibacterial treatment is not adequate, and multidrug resistance to it is developing. Henceforth, the antibacterial effect of the ethanolic extract of *Nepeta deflersiana* was put to test against red-complex bacteria in patients with chronic periodontitis. Methods: Well diffusion and micro broth dilution procedure by Alamar blue were applied to assess the zone of inhibition (ZOI), the minimum inhibitory concentration (MIC), and the minimum bactericidal concentration (MBC). Anti-virulence efficacies of the plant extract that comprise of adherence and formation of biofilms were examined by the process of adherence and biofilm production assay. Results: The crude extract of *Nepeta deflersiana* exhibited significant inhibitory outcome against periodontopathic bacteria with noteworthy MIC (0.78–3.12 mg/mL), inhibitory zone (12–20 mm), as well as MBC (3.12–12.50 mg/mL). The *N. deflersiana* extract inhibited bacterial adhesion ranging from 41% to 52%, 53% to 66%, and 60% to 79% at the given MIC × 0.5, MIC × 1, and MIC × 2 in succession. Substantial suppression was also developed in the biofilm production of the investigated periodontopathic strains following exposure to numerous concentrations of *N. deflersianan* extract for a period of 24 and 48 h. Conclusion: These outcomes divulge a new concept that *N. deflersiana* extract can be utilized to manufacture valuable antibacterial compounds to treat chronic and acute periodontitis. This identifies *N. deflersiana* as an essential natural source for future drug development.

## 1. Introduction

Periodontitis is recognized as the second most common infection globally after dental deterioration. It is elicited by the formation of a complex biofilm of microbes in the subgingival region, which attach to host cells and tissues, leading to continuous injury and destruction of the tooth-supporting apparatus [1,2,3]. The species of bacteria belonging to two major complexes (orange and red complexes) are thought to be etiologic agents of periodontal diseases [4]. The red complex bacteria, which consists of *Treponema denticola (T. denticola)*, *Tannerella forsythia (T. forsythia),* and *Porphyromonas gingivalis (P. gingivalis),* is the most significant complex existing together in the subgingival plaque next to the periodontal pocket epithelial lining in deep areas and plays a major collaborative role in the progression of periodontal disease [1]. Animal researches have also reported the coordinated and collaborative pathogenicity of combined infections with *Fusobacterium nucleatum (F. nucleatum)*, *T. denticola*, *T. forsythia* and *Aggregatibacter actinomycetemcomitans (A. actinomycetemcomitans), and P. gingivalis* [5,6].

Prevention of dental plaque accumulation represents a practical approach in the control of periodontal diseases. Previous studies by researchers worldwide have documented the role of various herbal preparations as antimicrobial agents with considerable therapeutic activity on the reduction of bacterial counts and dental plaque formation. Furthermore, the effect of polyherbal mixtures on the reduction of microbial count and plaque accumulation has been reported [7].

In recent years, *Nepeta deflersiana (N. deflersiana),* locally known as “mokerker alkotat”, a popular plant with medicinal value in Saudi Arabia, has been utilized as an antioxidant, anti-inflammatory, antimicrobial, antirheumatic agent, and carminative in folk medicine [8,9]. Nepeta, belonging to the Labiate family, is an aromatic herb, which comprises around 250 species and is found in the Middle East, Asia, and some parts of Europe [10]. *N. deflersiana* has recently gained considerable attention as an antimicrobial agent. However, there are very few studies available in the literature on *N. deflersiana* plant extract’s antibacterial activity.

In our previous studies, an attempt was made to appraise the synergistic and antibacterial properties of the plant extract of *N. deflersiana* in opposition to Gram-negative and Gram-positive pathogenic bacteria. The plant extract showed antibacterial activity against the reference strain of *Streptococcus pyogenes*. The synergy study also explained the significant bacteriostatic effect of *N. deflersiana* extract on *Staphylococcus epidermidis* and *Staphylococcus aureus* [11]. Furthermore, we also found significant antimicrobial activity of the ethanol extract fraction of *N. deflersiana* against Gram-negative microorganisms [12].

However, at present, no evidence is available on the antibacterial activity of *N. deflersiana* on bacterial species that play a significant part in advancing periodontitis and are strongly connected with the clinical advancement of chronic periodontitis. Therefore, the current research seeks to analyse the role of *N. deflersiana* as an antimicrobial agent against bacterial species associated with red-complex bacteria (*T. denticola, T. forsythia,* and *P. gingivalis*) that propounds a part of the climax community in oral biofilms, in addition to *A. actinomycetemcomitans* from clinical isolates of patients with periodontal disease.

## 2. Results

### 2.1. Antibacterial Efficacy of N. deflersiana Extract

**Figure 1:**
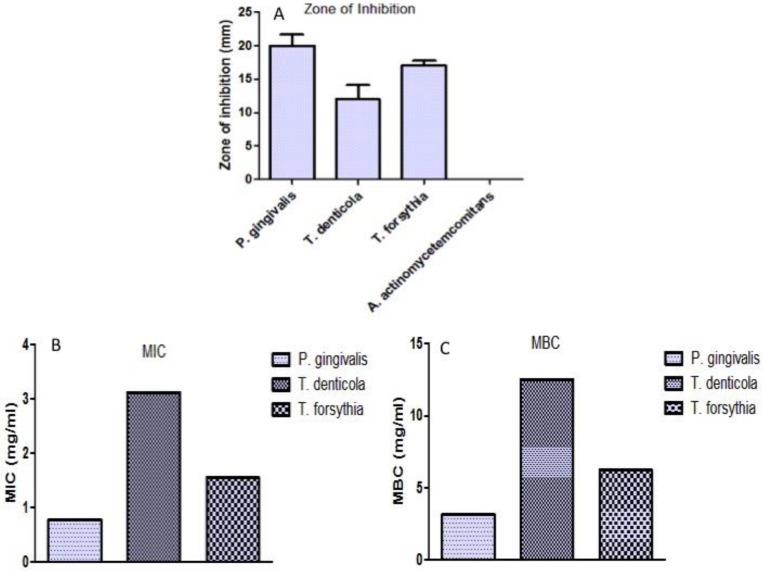
Effect of ethanolic extract of *N. deflersiana* against periodontal bacteria. (**A**) Zone of inhibition formed by *N. deflersiana* extract against *P. gingivalis, T. forsythia,* and *T. denticola.* (**B**,**C**) MIC and MBC values of *N. deflersiana* extract against *P. gingivalis, T. forsythia,* and *T. denticola.*

To establish the antibacterial efficacy of *N. deflersiana* extract, the red complex strains of bacteria causing periodontopathic infection such as *T. forsythia*, *P. gingivalis, T. denticola,* and *A. actinomycetemcomitans* were employed. The susceptibility assay exhibited that the extract has more antibacterial potency than *P. gingivalis,* whereas no antibacterial efficacy was found against *A. actinomycetemcomitans* (Figure 1). A more than 8-mm zone size was deliberated as being significant regarding the susceptibility of the tested strains of bacteria to *N. deflersiana* extracts examined. Beyond 8-mm zone size, the bacterial strains were found to be exposed to known concentrations of the extracts, in an effort to identify MIC and MBC.

To identify MIC and MBC values, the designated strains of bacteria were treated with the concentration mentioned above of the extract and subsequently incubated for 24 h. The minimum concentration of the *N. deflersiana* extract with no change in Alamar blue colour was considered as MIC. Figure 1 showed that bacterial strains *T. forsythia*, *P. gingivalis,* and *T. denticola* were considerably sensitive to the extract. The growth of bacteria was repressed with MIC and the MBC ranging from 0.78 to 3.12 mg/mL and 3.12 to 12.50 mg/mL, respectively. Such outcomes were supported with subsequent excellent inhibitory zone ranging from 12 to 20 mm.

### 2.2. Inhibition of Bacterial Adhesion by N. deflersiana

**Figure 2:**
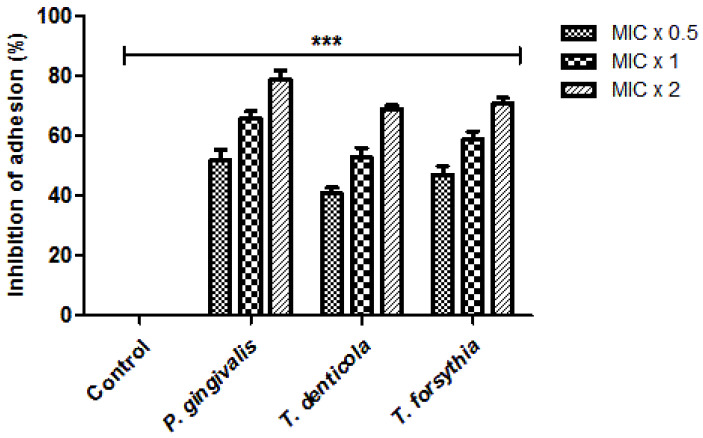
*N. deflersiana* extract reduces bacterial adhesion. Adhesion assay based on Alamar blue was employed to assess the efficacy of *N. deflersiana* on *T. forsythia*, *P. gingivalis*, and *T. denticola* adherence. Each bacterial strain were treated with MIC × 0.5, MIC × 1 and MIC × 2 values of the plant extract at 37 °C for 6 h. Control bars specify each untreated bacterial strain, revealed as 0% inhibition. The data was conferred from three independent investigations using means ± SD *** *p* < 0.0001.

The adhesion process was performed by utilising Alamar blue dye in order to evaluate the efficacy of *N. deflersiana* on the examined adherence of the bacteria. It was seen that bacterial cells, when treated with varying concentrations of the extract, showed unpredictable bacterial adherence inhibition, stipulating that the consequence was concentration-dependent (Figure 2). For bacterial strains such as *T. forsythia, T. denticola,* and *P. gingivalis,* the plant extract was proficient enough to inhibit the adhesion of bacterial strains ranging from 41% to 52%, 53% to 66%, and 60% to 79% at MIC × 0.5, MIC × 1 and MIC × 2, respectively. The tested bacterial strains with zero concentration of extract were contemplated as control. The findings suggested that the *N. deflersiana* plant extract was sensitively proficient in diminishing bacterial strains’ adhesion, even at concentrations considered subinhibitory. 

### 2.3. Inhibitory Property of Bacterial Biofilm Formation by N. deflersiana

**Figure 3:**
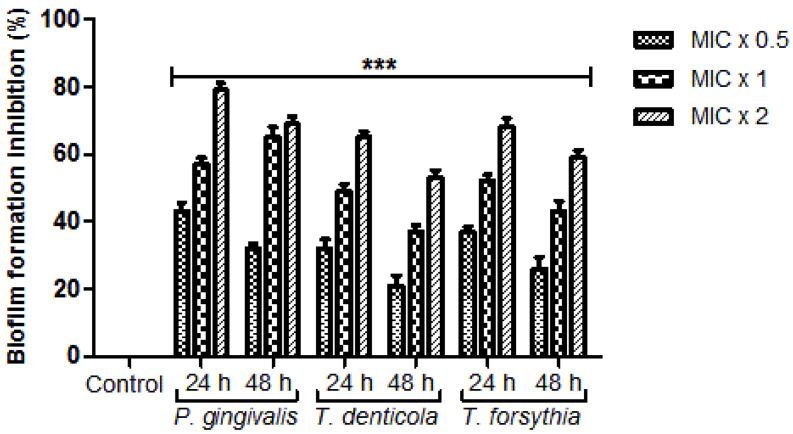
*N. deflersiana* extract diminishes biofilm formation. *P. gingivalis, T. forsythia, and T. denticola* were incubated with MIC × 0.5, MIC × 1, and MIC × 2 values of *N. deflersiana* extract in biofilm-producing environments for 24 and 48 h. Control bars specify each untreated bacterial strain, revealed as 0% inhibition. The data is conferred from three independent investigations using means ± SD *** *p* < 0.0001.

Remarkably, significant inhibition of the biofilm formation was noticed in every examined bacterial strain treated with numerous concentrations of *N. deflersiana* extract for 24 h and 48 h (Figure 2). Subsequently, after 24 h, at varying concentrations of MIC × 0.5, MIC × 1, and MIC × 2 of the analytic entities, the inhibition of biofilm formation to *T. forsythia, P. gingivalis,* and *T. denticola* was determined in the range of 31% to 43%, 49% to 57%, and 65% to 79%, respectively (Figure 3). In contrast, after 48 h, the suppression of biofilm formation at the various MIC × 0.5, MIC × 1, and MIC × 2 was determined in the range of 21% to 32%, 37% to 69%, and 53% to 65%, respectively (Figure 3). The tested bacterial strains with zero concentration of the plant extract were contemplated as the control. Additionally, the rate of inhibition of the formation of the biofilm by the *N. deflersiana* extract was established based on concentration and treatment time. The detections revealed that the plant extracts remarkably diminished biofilm formation in the examined bacterial strains. 

### 2.4. Effect N. deflersiana Extract on Bacterial Growth 

**Figure 4:**
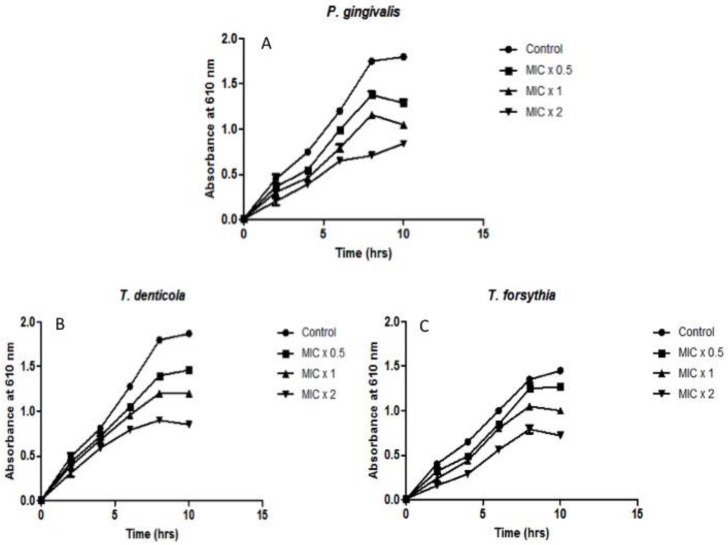
Effect of *N. deflersiana* on bacterial growth kinetics. Illustrative bacterial strains of (**A**) *P. gingivalis*, (**B**) *T. denticola,* and (**C**) *T. forsythia* were treated with different concentrations (MIC × 0.5, MIC × 1 and MIC × 2) of ethanolic extract of *N. deflersiana*. Untreated bacterial growth cycle was considered as growth control. The absorbance was measured at 610 nm at five-time intervals of 2 h. The data was conferred from three independent investigations using means ± SD (*p* < 0.0001).

The real-time analysis of *N. deflersiana* extract on the growth of bacteria at various time intervals was conducted. To define the time killing kinetic assay, 180 μL of bacterial culture with an absorbance of 0.01 at O.D._610_ was exposed to the extract (20 μL) at various concentrations (MIC × 0.5, MIC × 1, and MIC × 2). Bacterial growth was detected at intervals of 2 h (Figure 4). It is self-evident that bacterial growth declined upon treatment of the extract at various concentrations. The time killing kinetic specified that the bactericidal activity on the examined bacterial strains depends on the extract’s doses. Our result distinctly highlights the strong antibacterial effect of *N. deflersiana* against various pathogenic perio-pathobionts.

## 3. Discussion

Oral health is considered essential for overall general health. Oral diseases are long-standing polymicrobial encounters to host tissues, which, in specific situations, and a particular part of the populace, guide the damage of the hard and soft tissue on all sides of the teeth, personifying periodontitis disease. More than 700 different microbial species exist in periodontitis, most of which have been shown to play an essential role in starting and developing periodontitis [13,14]. Periodontopathic microbes such as *T. denticola*, *A. actinomycetemcomitans*, *P. gingivalis,* and *T. forsythia* comprise the most important pathogens in periodontitis [15]. 

Thus, in this study, we studied the antimicrobial properties of ethanolic extracts of *Nepeta deflersiana* on oral plaque bacteria comprising *A. actinomycetemcomitans, T. forsythia, T. denticola,* and *P. gingivalis.* In the current study, the ethanolic extract of *Nepeta deflersiana* was first tested for its antimicrobial activity against oral plaque bacteria *A. actinomycetemcomitans*, *P. gingivalis*, *T. forsythia,* and *T. denticola*. The ethanolic extract of *N. deflersiana* exhibited antibacterial action against the examined pathogenic flora in the periodontal region, except *A. actinomycetemcomitans,* displaying the MIC as MBC, ranging from 0.78–3.12 mg/mL and 3.12–12.5 mg/mL, respectively. It was noticed in the present study that *N. deflersiana* indicated significant antibacterial efficacy against all the tested pathogenic periodontal flora, except for *A. actinomycetemcomitans* (Figure 1). The antibacterial efficacy of the tested plant extract was observed to be maximum against *P. gingivalis* (MIC = 0.78 mg/mL, MBC = 3.12 mg/mL), and minimum against *T. denticola* (MIC = 3.12 mg/mL, MBC = 12.5 mg/mL). In support of this statement, it was detected from the earlier phytochemical scientific literature that compounds of *Nepeta deflersiana* are recognized as having antiviral, antifungal, antibacterial, and anti-inflammatory properties [16]. This study’s outcomes are reinforced by another bacteriological study in which it was described that ethanolic extract *Nepeta deflersiana* has a significant bactericidal effect on pathogenic Gram-positive bacteria [17]. 

In the current study, the bacterial adhesion property of the *N. deflersiana* extract was expressively productive in reducing examined bacterial adhesion, even when tested at subinhibitory concentrations compared to the control, where no plant extract was used (Figure 2). Similarly, the rate of biofilm formation was significantly reduced in the tested bacterial strains when they were exposed to the *N. deflersiana* plant extract for 24 and 48 h at different concentrations (Figure 3). Concerning bacterial growth, our study showed that bacterial growth was reduced according to treatment times with varying concentrations of the plant extract. The time killing kinetic suggested a dose-dependent bacterial strain growth reduction on tested bacteria when exposed to *N. deflersiana* plant extract. This result suggests that the extract has a strong antibacterial activity against pathogenic red-complex oral plaque bacteria.

There is no or very little data available in the literature regarding *N. deflersiana* plant extract’s effect on oral plaque bacteria. Only one study investigated the essential oil of *N. deflersiana* for its antiviral activity with regard to the avian influenza virus—it depicted moderate activity of the oil on the influenza virus strain of H5N1. The same study found that *Nepeta* oil showed good activity against respiratory tract pathogenic bacteria [17]. Similar results were found in a study on Gram-positive bacteria with *N. deflersiana* extract, suggesting that the potential toxicity of its active ingredients needs further evaluation [11].

The development of innovative antibacterial agents is inadequate because of insufficient documentation on the drug targeting bacteria [16]. These particular characteristics exaggerate the drug resistance development of current antimicrobial agents and highlight the need for novel antimicrobial drug development with several target sites. Based on these shreds of evidence, the present study was focused on leading virulence factors in *T. denticola, A. actinomycetemcomitans, T. forsythia, and P. gingivalis,* such as biofilm formation and adherence. Consequently, indicating the virulence factors may be an innovative model in the evolution of unique and fruitful antimicrobial options for periodontal pathogenic flora.

Bacterial virulence properties are related to their ability to adhere to host tissue, resulting in biofilm formation. This research also exposed that *N. deflersiana* inhibited the particular adhesion as well as biofilm formation. The adherence features are unswervingly linked with biofilm formation and therefore expedite the genesis of dental plaque [18]. Bacterial cells of the red complex have a specific type of protein known as adhesins, which help in the adhesion of the bacteria on the host cell surface [19]. Glycoproteins are encoded on the host cell surface by adhesins of the periodontal flora. This was discernible from conclusions of this report: the ethanolic type plant extract of *N. deflersiana* expressively repressed adherence in *T. denticola*, *T. forsythia,* and *P. gingivalis* in a dose-dependent manner ranging from 41% to 79%.

Moreover, the adherence of *T. denticola, P. gingivalis,* and *T. forsythia* to host cells lead to biofilm formation. The biofilm is a vibrant factor of virulence in periopathodontic bacteria, increasing bacterial resistance to the greatest conventional antimicrobial drugs [20,21]. Besides, plenty of publications have divulged that bacterial biofilms are highly resistant compared to planktonic cells in current antibacterial therapy. Biofilm production in the salivary pellicle provides a route for the development of dental plaque [22]. In this particular investigation, it was detected that the plant extract significantly diminished biofilm production in a dosage-dependent method, between 32% to 79% and 21% to 69% after a treatment period of 24 h and 48 h, respectively. The consequences of the present study assist in accepting that *N. deflersiana* particularly targets adhesive-type proteins of the membrane, which support adherence, therefore inhibiting host tissue adherence while inevitably inhibiting the genesis of biofilms. Thus, the present study’s results are indispensable and encouraging with regard to this novel antibacterial agent’s effect on red-complex oral plaque bacteria. Moreover, it is economically beneficial as *N. deflersiana* is a local flora easily available and may give rise to pioneering of novel drugs.

## 4. Materials and Methods

### 4.1. Preparation of the Extract

The aerial part of the *N. deflersiana* plant obtained from Abha, Saudi Arabia, was identified by a plant taxonomist from the Biology Department, King Khalid University, Abha, Saudi Arabia, for accuracy and genuineness. The plant sample was kept at the Herbarium of Biology Department for future reference to procure a voucher number (#48658). The plant was then ground to a powder for 10 s. 50 gm of the powdered substance was placed in a muslin cloth and put into a Soxhlet extractor for uninterrupted hot extraction with absolute ethanol for a period of 72 h. Subsequently, the ethanolic extract of *N. deflersiana* was subjected to filtration, and the filtrate was evaporated at a reduced temperature and pressure with the Buchi R-200 Rotavapor. The percentage yield of the plant extract was computed as per the dried substance—16.86% *w*/*w*. The dried extracts were also dissolved at a concentration of 0.2 g/mL in ethanol and employed for antimicrobial susceptibility. Furthermore, stock solutions were made, and an eventual volume was accomplished by diluting the stock to 2-fold dilution, ranging from 0.2 g/mL–50 μg/mL, which was employed later to identify MIC and MBC values.

### 4.2. Specimen Collection and Isolation of Bacteria

Collection of plaque samples was done from patients with inflammation in periodontal cells. The plaque samples were collected from patients diagnosed as generalized periodontitis stage III grade B. The diagnosis was based on the distribution and severity of periodontal attachment loss in relation to teeth present in the oral cavity [23]. Plaque samples were accumulated by infusing Gracey-curette (Hu-Friedy, Chicago, IL, USA). The plaque samples were collected as the curette touched the base of the periodontitis region without any damage to adjacent tissues. The plaque samples were moved to an anaerobic transport media, Sodium thioglycolate (Sigma-Aldrich, Dermstadt, Germany).

Selective media was used for the isolation of *P. gingivalis, T. denticola, A. actinomycetemcomitans,* and *T. forsythia* [24], incubated anaerobically with nitrogen content of 80%, hydrogen content 10%, and carbon dioxide content 10% for a period of 3 to 7 days at a temperature of 37 °C. Further, the growth of the desired bacteria on the particular media was recognized based on morphology of the colony, and species identification was done by biochemical tests.

### 4.3. Utilization of the Well Diffusion Method for Antibacterial Susceptibility Assay

Growth of the bacterial strains was done in logarithmic period (O.D._610_ of 0.4 to 0.6) inside a lysogenic broth (LB) broth for evaluation of the tested bacterial strains to particular susceptibility to the *N. deflersiana* plant extract. Subsequent dilution of the bacterial strains was done in LB broth up to O.D._610_ of 0.01. Antibacterial efficiency of the plant extract was evaluated by the agar well diffusion process [25]. Concisely, 6-mm wide wells were created in LB agar with the cap of a sterilized syringe, and the sterile swab was used to form a lawn culture from the diluted culture on the agar plate. 20 μL of the extract (0.2 g/mL) were shifted in the triplicate wells of the petri dish and additionally incubated anaerobically at 37 °C for 24 h. The width of the inhibition zone of microbial growth containing the width of the well was computed in millimeters. Authentic inhibition zone was evaluated by subtracting the average inhibition zone by the plant extract from the average inhibition zone by ethanol.

### 4.4. MIC and MBC Determination

The MIC and MBC determination of the crude extract from *N. deflersiana* was done with some modification [26]. The concentrations of *N. deflersiana* extract utilized for determinations of MICs and MBCs on chosen strains of bacteria were employed with 2-fold dilution ranging from 0.2 g/mL up to 50 μg/mL. To define the MIC, the selected strains having O.D._610_ of 0.4–0.6 were further subjected to dilution to a hypothetical O.D._610_ of 0.01 in LB broth. Consequently, 180 μL of culture from all the strains were poured inside the wells consisting of sterile polystyrene flat-bottom 96-wells plate. Then, 20 μL from the extract of 2-fold dilution were transferred to the triplicate wells, and 20 μL of 5% ethanol was transferred to the triplicate wells studied as control.

Additionally, it was anaerobically incubated for 24 h at 37 °C. 20 μL of Alamar blue dye (Thermo Fisher, MA, USA) was transferred inside all wells after an incubation of 24 h, and the pink colour change was monitored hourly. The least concentration of *N. deflersiana* inside a well that was unable to change the Alamar blue colour was deliberated as MIC. 

To examine the MBC of the *N. deflersiana* extract against the tested bacterial strains, 10 μL culture was taken from those wells where the Alamar blue colour did not change, was sub-cultured on LB agar plate, and subsequent anaerobic incubation was done at 37 °C for a period of 24 h. The least concentration of *N. deflersiana* extract with no bacterial growth observed was studied as MBC.

### 4.5. Adherence Assay

Adherence assay was accomplished by the technique elucidated previously [27]. Briefly, 100 μL of examined bacterial inoculum with absorbance 0.01 at O.D._610_, cultured in the RPMI 1640 medium, and subsequently buffered at neutral pH through 0.165 M Mops (morpholinepropanesulphonic acid) was transferred to the 96 wells plate. Consequently, the bacteria cells were exposed under various concentrations (MIC × 0.5, MIC × 1, MIC × 2) of the plant extract and anaerobically incubated at 37 °C for a period of 6 h. Untreated cells of the bacteria were utilized in all phases of examination as a negative control. Therefore, the incubated medium was relinquished, and rinsing of all the wells was done with double 200 μL PBS to eradicate bacterial cells that were non-adherent. 100 μL of Alamar blue at 5% concentration in RPMI 1640 medium were transferred into each well and anaerobically incubated for 6 h at 37 °C. Fluorescence signs were read at 555Ex/585Em using an HT microplate absorbance reader (BioTek Instruments, Winoosky, VT, USA).

### 4.6. Formation of Biofilms

Biofilm formation inside the sterilized 96 wells flat-bottom plates was achieved by inoculating the examined suspensions of bacterial inoculum (O.D._610_ of 0.01) grown in a media of RPMI 1640 media buffered at neutral pH with 0.165 M Mops and anaerobically incubated at 37 °C for a period of 6 h [28]. After an adhesion period of 6 h, the media was taken out prudently with no disruption in biofilm formation. Then, various concentrations of the plant extract (MIC × 0.5, MIC × 1, MIC × 2) were prepared in RPMI 1640 medium and transferred into the 96 wells plate. The bacterial cells without any treatment in all phase of examination were considered as a negative control. Additionally, the 96 wells plate was incubated in an anaerobic environment for 24 h and 48 h at 37 °C. Evaluation of the efficiency of the examined *N. deflersiana* extract on biofilm formation was done as explained [29].

### 4.7. Time Killing Kinetic Assay

To explore *N deflersiana* extract’s efficacy on the examined bacterial cells, 180 μL of the bacterial culture with 0.01 absorbance at O.D._610_ was exposed to 20 μL of the extract at various concentrations (MIC × 0.5, MIC × 1, MIC × 2). The culture well having 20 μL of 5% ethanol was studied as the control. Additionally, the 96 well plates were incubated in an anaerobic environment at 37 °C, and the absorbance was calculated at 610 nm at the five intervals of 2 h. The average calculated absorbance was graphed over time.

### 4.8. Ethical Consideration

Ethical approval was taken from the Scientific Research Committee, College of Dentistry, King Khalid University (SRC/ETH/2016-17/053). Full privacy and confidentiality was given to the participants.

### 4.9. Statistical Analysis

All the investigations were conducted thrice and the data divulged as mean ± SD. Statistics were accomplished by GraphPad prism software-6.0 (La Jolla, San Diego, CA, USA). Two-tailed Student’s t-test was achieved to observe the differences between the groups and *p* < 0.0001 (***) were considered significant. 

## 5. Conclusions

It is widely known that plant compounds have antibacterial properties. As antibiotic resistance by most pathogens is increasing, the diminishing capacity of currently available compounds has highlighted plant constituents as an alternative source of new classical compounds to overcome and fight bacterial infections. This study shows that the *N. deflersiana* extract possesses antimicrobial efficacies with great effectiveness. Compounds that are produced by plants may affect microbes, either killing them or suppressing their growth with less or no harm to the host. These findings accentuate plants’ crucial role in the development of future drugs. 

## Data Availability

The data of this study is contained within the article or supplementary material. The data presented in this study are available in this manuscript.
